# Airport noise disturbs foraging behavior of Japanese pipistrelle bats

**DOI:** 10.1002/ece3.8976

**Published:** 2022-06-12

**Authors:** Weiwei Wang, Huimin Gao, Chengrong Li, Yingchun Deng, Daying Zhou, Yaqi Li, Wenyu Zhou, Bo Luo, Haiying Liang, Wenqin Liu, Pan Wu, Wang Jing, Jiang Feng

**Affiliations:** ^1^ 85112 Jilin Provincial Key Laboratory of Animal Resource Conservation and Utilization Northeast Normal University Changchun China; ^2^ 56714 Key Laboratory of Southwest China Wildlife Resources Conservation of Ministry of Education China West Normal University Nanchong China; ^3^ 85112 College of Life Science Jilin Agricultural University Changchun China

**Keywords:** airport noise, bat, foraging behavior, noise pollution

## Abstract

The expansion of anthropogenic noise poses an emerging threat to the survival and reproductive success of various organisms. Previous investigations have focused on the detrimental effects of anthropogenic noise on the foraging behavior in some terrestrial and aquatic animals. Nevertheless, the role of airport noise in impairing foraging activities of most wild animals has been neglected. Here, we aimed to assess whether foraging behavior in free‐living Japanese pipistrelle bats (*Pipistrellus abramus*) can be disturbed by airport noise. We used audio recording to monitor foraging activities of bats at 11 sites around the runway of a municipal airport. We quantified noise level and spectra, aircraft activity, habitat type, nightly temperature, wind speed, and moon phase for each site. The analysis revealed that noise level and aircraft activity were significant negative predictors for the number of bat passes and feeding buzzes around the runway, even after controlling for the effects of other environmental factors. There was no marked spectral overlap between bat echolocation pulses and airport noise in the presence and absence of low‐flying aircraft. The spectro‐temporal parameters of echolocation vocalizations emitted by bats were dependent on noise level, aircraft activity, and habitat type. These results provide correlative evidence that airport noise can reduce foraging activities of wild pipistrelle bats. Our findings add to the current knowledge of adverse impacts of airport noise on foraging bats in artificial ecosystems and provide a basis for further research on the mechanisms behind noise pollution near airports.

## Graphical abstract

We test the relationship between airport noise and foraging behavior in wild bats. The airport noise disturbs foraging activities of bats. Echolocation pulse parameters of bats are predicted by noise level and aircraft activity.

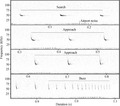

## 1 INTRODUCTION

The extent and intensity of anthropogenic noise have rapidly increased with the development of urbanization, transportation networks, and resource extraction, leading to serious environmental pollution of global concern (Barber et al., 2010; Buxton et al., 2017; Shannon et al., 2016). It has been shown that noises from trucks, cars, and motorcycles can be louder than 50 dBA around roads in urban ecosystems during the day and night (Barber et al., 2010; D’Alessandro & Schiavoni, 2015). In most rural and natural ecosystems, human‐induced noise doubles the level of background noise, inducing a marked shift in the patterns of the soundscape (Buxton et al., 2017; Iglesias‐Merchan et al., 2021). Adverse effects of anthropogenic noise on health and other fitness components have been demonstrated in a variety of organisms (Barber et al., 2010; Jerem & Mathews, 2021; Kleist et al., 2018). In terms of humans, for example, chronic noise exposure can cause multiple health issues ranging from hearing loss and communication handicaps to sleep deprivation and cardiovascular disease (Barber et al., 2010; Giles‐Corti et al., 2016). For wild animals, human‐induced noise can lower their foraging efficiency (Francis et al., 2009), retard social information transfer (Mason et al., 2016), weaken anti‐predator defenses (Patricelli & Blickley, 2006; Slabbekoorn & Ripmeester, 2008), and reduce reproductive success (Senzaki et al., 2020). Furthermore, the impacts of anthropogenic noise are not confined to individuals or populations of single species but also reach the community level (Herrera‐Montes & Aide, 2011; Kleist et al., 2018). Consequently, noise pollution induced by human activities has been a focus of global change research in the 21st century (Francis & Barber, 2013; Gomes et al., 2021; Jerem & Mathews, 2021; Kleist et al., 2018; Simpson et al., 2016).

Municipal airports are one of the most common sources of anthropogenic noise due to the presence of multiple vehicles, including commercial aircraft, luggage vans, and cars (Mato & Mufuruki, 1999). The periods of airport noise are highly repeatable and predictable, with a sudden peak while aircraft takeoff and land. It has been estimated that the noise level at airports ranges from 55 to 110 dBA, and the noise spectrum varies from 0.05 to 20 kHz, depending on the airport scale, the number of flights, and the type of aircraft (Ozkurt, 2014; Sari et al., 2014; Zaporozhets et al., 2011). Previous investigations have verified that airport noise affects daily activities of some birds and mammals via interfering with auditory perception (Klett‐Mingo et al., 2016; Weisenberger et al., 1996). In particular, great tits (*Parus major*) increased their vigilance time when exposed to loud aircraft noise, resulting in a decline in feeding effort (Klett‐Mingo et al., 2016). European blackbirds (*Turdus merula*) and some other songbirds near airports advanced the onset of the dawn chorus to avoid temporal overlap with the peak period of aircraft activity (Gil et al., 2015; Sierro et al., 2017). Captive mule deer (*Odocoileus hemionus*) and mountain sheep (*Ovis canadensis*) showed increased vigilance and rapid heart rates in response to simulated aircraft noise, albeit with the sign of habituation after long‐term noise exposure (Weisenberger et al., 1996). Nonetheless, the potential effects of airport noise on foraging behavior in most wild animals remain an open question.

Bats provide an alternative model to disentangle the harmful effects induced by noise pollution. Although many bats exploit underground roosts for sleep and social interactions, they may suffer from noise‐induced stress during the day due to human recreation, limestone quarrying, and introduction of transportation corridors (Ancillotto et al., 2019; Geipel et al., 2019; Wu et al., 2018). Bats also experience a high risk of noise disturbance during evening emergence and foraging, given that their activity time overlaps with the aircraft and other vehicles at night (Luo et al., 2021; Speakman, 1991). Most echolocating bats rely primarily on vocalizations for spatial orientation and prey detection through emitting echolocation pulses and listening for the returning echoes from objects (Fenton, 2013; Schnitzler et al., 2003). In addition to the use of echolocation, some gleaning bats also seek foraging targets by listening for rustling sounds generated by prey movements (Denzinger & Schnitzler, 2013; Geipel et al., 2013). Echolocating bats suffer from the sensorial challenge of acoustic masking if anthropogenic noise blocks prey echoes or sound cues generated by prey (Fenton, 2013; Siemers & Schaub, 2011). Previous controlled experiments in the laboratory revealed that greater mouse‐eared bats (*Myotis myotis*) tended to avoid foraging in a noisy environment, and playback of traffic noise from the highway caused a decrease in feeding efficiency (Siemers & Schaub, 2011). Experimental manipulation of road traffic noises below and above 20 kHz consistently impaired feeding activities in Daubenton’s bats (*Myotis daubentonii*), common pipistrelles (*Pipistrellus pipistrellus*), and soprano pipistrelles (*Pipistrellus pygmaeus*), suggesting that anthropogenic noise serves as an aversive stimulus for foraging bats irrespective of the magnitude of spectral overlap with ultrasonic pulses and prey echoes (Finch et al., 2020; Luo, Siemers, et al., 2015). Field monitoring showed that foraging activities in Brazilian free‐tailed bats (*Tadarida brasiliensis*) were reduced by 40% at noisy compressor sites as compared to quieter well pads, albeit this was not the case for California myotis (*Myotis californicus*), little brown bats (*Myotis lucifugus*), or canyon bats (*Parastrellus hesperus*) (Bunkley et al., 2015). Le Roux and Waas (2012) found that New Zealand long‐tailed bats (*Chalinolobus tuberculatus*) exhibited no marked difference in flight activities during the presence and absence of aircraft, and playback of simulated aircraft noise had weak effects on their flight activities versus silent controls (Le Roux & Waas, 2012). These findings indicate that the impacts of anthropogenic noises on foraging‐related activities in bats may differ by species and noise types, thus warranting further investigation.

The goal of this study was to assess the relationship between airport noise and foraging behavior in wild Japanese pipistrelle bats (*Pipistrellus abramus*). Japanese pipistrelle bats are aerial foragers that capture night‐active insects near residential areas, rivers, and the edges of forests (Fujioka et al., 2014; Motoi et al., 2017). They usually dwell in the crevices of man‐made structures, and initiate foraging activities within 30 min of local sunset (Shao et al., 2014). The dietary composition of *P*. *abramus* is dominated by Diptera and Hymenoptera (Motoi et al., 2017). These bats emit frequency‐modulated echolocation pulses, with the highest energy on the first harmonic. The first harmonic of echolocation pulses emitted by *P*. *abramus* varies from 40 to 95 kHz, depending on behavioral context and geographic location (Hiryu et al., 2008; Ma et al., 2010; Shao et al., 2014). Our previous survey has shown that some *P*. *abramus* forage in open and edge habitats around the runway of a municipal airport in China (Wang et al., 2019). The foraging activities of *P*. *abramus* at the airport overlap largely with nighttime flight time of various aircraft, putting the bats at a high risk of exposure to intense noise. We hypothesized that airport noise could disturb the foraging behavior of *P*. *abramus* following previous studies (Bunkley et al., 2015; Luo, Siemers, et al., 2015). To test our hypothesis, we monitored bat passes and feeding buzzes as a function of noise level, aircraft activity, habitat type, nightly temperature, wind speed, and moon phase around the runway of an airport. We quantified the relationship among noise level, aircraft activity, and spectro‐temporal parameters of echolocation pulses emitted by foraging bats. We made the following predictions: (1) the number of bat passes and feeding buzzes would be negatively associated with noise level and aircraft activity at the airport; and (2) spectro‐temporal parameters of echolocation vocalizations in foraging bats would show no marked change with increased level of airport noise if noise spectrum was not within the frequency range of ultrasonic pulses.

## 2 MATERIALS AND METHODS

### 2.1 Study areas

During April and July 2020, we conducted field surveys at Gaoping Airport in Nanchong city, China. Gaoping Airport is located about 10 km from the center of the city and covers an area of nearly 210 hectares. The length and width of active runway were 2.8 and 0.06 km, respectively. On average, aircraft takeoff and land up to 28 times per day, and more flights are being planned following the enlargement of the terminal (http://data.carnoc.com/corp/airport/nao.html). The airport and surrounding area contain various microhabitats suitable for insect exploitation by *P*. *abramus*, including some grasslands, coniferous forests, residential zones, and ponds. Experimental procedures complied with the relevant laws of China for studies involving vertebrates and the ASAB/ABS Guidelines for the Use of Animals in Research. Fieldwork was approved by the authorities of Gaoping Airport and by the committee on the Use and Care of Animals of the China West Normal University (approval number: CWNU20210002).

### 2.2 Monitoring of bat activities and environmental factors

**FIGURE 1:**
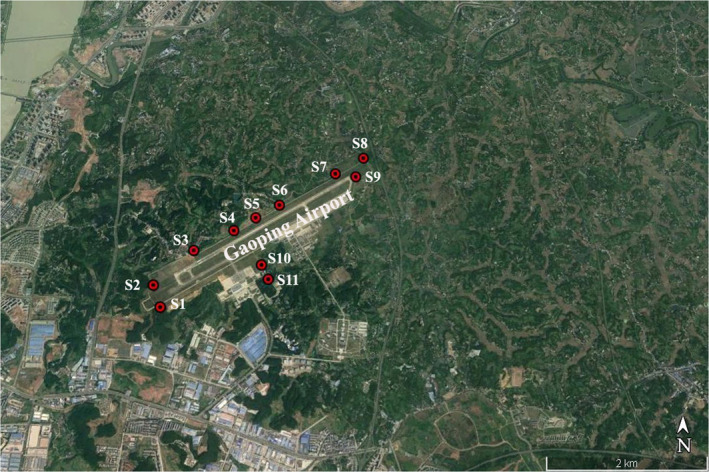
Geographic locations for sampling sites at Gaoping Airport

We monitored foraging activities in *P*. *abramus* among 11 sampling sites around the runway (Figure 1). The straight‐line distance between different sampling sites was 0.50–2.76 km. We recorded echolocation vocalizations given by foraging *P*. *abramus* using an ultrasonic sound acquisition system (UltraSoundGate 116, Avisoft Bioacoustics, Berlin, Germany) connected to a laptop computer, with a sampling frequency of 375 kHz at 16 bits/sample. An ultrasonic microphone (UltraSoundGate CM16, Avisoft Bioacoustics, Berlin, Germany) was held 1.7 m above the ground and was oriented toward the night sky at an angle of 45°. We conducted acoustic recordings across all the sites from 19:30 to 22:30 on 10 nights, covering the peak period of feeding activities in *P*. *abramus*. At each site, acoustic sampling lasted for 5 min per night and was repeated 10 times in random order after an interval of 4–5 days. After excluding the time for commuting between sites by electric bicycles (Junzheng Network Technology Co., Ltd., Shanghai, China), the total recording time across 11 sites was 55 min per night. We measured the noise level at each site per 30 s during acoustic sampling using a sound‐level meter with A‐weighting mode (AR854, Shanghai Baoxin Instrument Co., Ltd., China). A‐weighting cuts off the frequencies outside the range of human hearing, which is widely used for quantifying the level of anthropogenic noise (Geipel et al., 2019; Shannon et al., 2016). We also observed the presence and absence of low‐flying aircraft upon monitoring the noise level.

We determined the habitat type for each site according to the degree of clutter (Kalko & Schnitzler, 1993). We defined a site as an edge space (*n* = 5 sites) if some trees occurred within 5 m of the site, and an open space (*n* = 6 sites) if there was no tree cover around the site. We obtained information on nightly temperature, wind speed, moon phase, and light level for each site, given that these factors may also influence echolocation and foraging behaviors of *P*. *abramus*. The nightly temperature and wind speed were extracted from Gaoping weather station located at ~6 km from the airport, using R package RNCEP (Kemp et al., 2012). The moon phase each night was obtained using the package lunar based on experimental date and geographic location of sampling sites (Lazaridis, 2014). For each site, light level was measured by an SW582 illuminance meter (Frank Electronics Co., Ltd., China) held horizontally at a height of 1.7 m. The light level at each site was approximately 0 lux during nighttime acoustic surveys and was excluded from further analysis. Upon finishing acoustic recordings, we caught two male *P*. *abramus* (Figure S1) at study sites using a mist net and hand net, confirming the reliability of species identification according to morphological and acoustic characteristics. The captured bats were released into their foraging areas after handling. The Chinese noctules (*Nyctalus plancyi*) were also monitored at one site during acoustic surveys, although this species was not included in this study due to a low number of echolocation vocalizations.

### 2.3 Sound processing

Sounds were visualized via the automatic procedure in Avisoft‐SASLab Pro (Avisoft Bioacoustics) based on 512 FFT, 100% frame size, and 87.5% temporal overlap. The first harmonic of echolocation pulses louder than 30 dB was used for analysis. Two acoustic indices were used to quantify foraging activities initiated by *P*. *abramus*, i.e., the number of bat passes and feeding buzzes. We assigned a bat as “pass” when two or more consecutive echolocation pulses were recorded following previous studies (Guo et al., 2021; Walsh & Harris, 1996). We divided echolocation behaviors of foraging bats into three sequences, namely search, approach, and terminal buzz phases (Ma et al., 2010). We applied five spectro‐temporal parameters to characterize bat echolocation pulses, including pulse duration, start frequency, end frequency, peak frequency, and bandwidth. Pulse bandwidth was calculated by subtracting end frequency from start frequency. Combined with field survey, we inspected whether bat echolocation vocalizations were emitted in the presence of low‐flying aircraft based on sound visualization and playback. To compare the difference in spectra between airport noise and bat echolocation vocalizations, we measured frequency parameters of airport noise in the presence and absence of low‐flying aircraft.

### 2.4 Statistical analysis

We computed the average noise level per night for each site during acoustic surveys. We applied a permutation test for two‐way analysis of variance (PANOVA) to examine the effects of experiment date and sampling site on foraging activities of bats. The Spearman correlation analysis was conducted to test the relationship between the number of bat passes and feeding buzzes. The generalized linear mixed model (GLMM) with a negative binomial distribution was employed to explore the relationship between environmental factors and foraging activities of bats across study sites. The linear mixed model (LMM) allowed us to assess the contributions of environmental factors to echolocation pulse parameters. The number of bat passes and feeding buzzes was entered into the GLMM as dependent variables. The noise level, aircraft activity, habitat type, nightly temperature, wind speed, moon phase, and their significant interactions based on likelihood ratio tests were entered into the GLMM as fixed variables. To fit the LMM, pulse duration, peak frequency, and bandwidth were assigned as dependent variables, whereas noise level, aircraft activity, other environmental factors, and their significant interactions were assigned as fixed variables. Prior to fitting the LMM, echolocation pulse parameters were Box–Cox transformed to approximate normality. For both the GLMM and LMM, experimental dates (*n* = 10) and feeding phases (*n* = 3) were assigned as random variables. The GLMM and LMM were conducted with the R packages glmmTMB (Brooks et al., 2017) and lme4 (Bates et al., 2013). We chose the best‐fitting GLMM and LMM (Table S1) based on Akaike’s information criterion corrected for small sample size (AICc) using the package MuMIn (Barton, 2016). All statistical analyses were performed in R 3.6.1 (R Foundation for Statistical Computing, Vienna, Austria). Data were given as mean ± SE.

## 3 RESULTS

### 3.1 Site variation in foraging activities of bats

A total of 585 bat passes were monitored during acoustic surveys, with an average of 5.32 ± 0.94 passes at each site per survey (Table S2). The number of bat feeding buzzes varied from 0 to 16 across all sites (Table S2). Despite weak effects of experimental dates (PANOVA: bat passes: *df* =9, *F* = 0.024, *p* = .80; feeding buzzes: *df* = 9, *F* = 0.25, *p* = .67), the number of bat passes and feeding buzzes exhibited pronounced differences at different sites (bat passes: PANOVA: *df* = 10, *F* = 9.41, *p* = .0018; feeding buzzes: *df* = 10, *F* = 20.15, *p* < .0001). Experimental dates and sampling sites showed no remarkable interactions (PANOVA: bat passes: *df* = 90, *F* = 0.012, *p* = .96; feeding buzzes: *df* = 90, *F* = 3.11, *p* = .080). There was a positive association between the number of bat passes and feeding buzzes (*r* = 0.69, *n* = 110, *p* < .0001).

### 3.2 Relationship between environmental factors and foraging activities of bats

**FIGURE 2:**
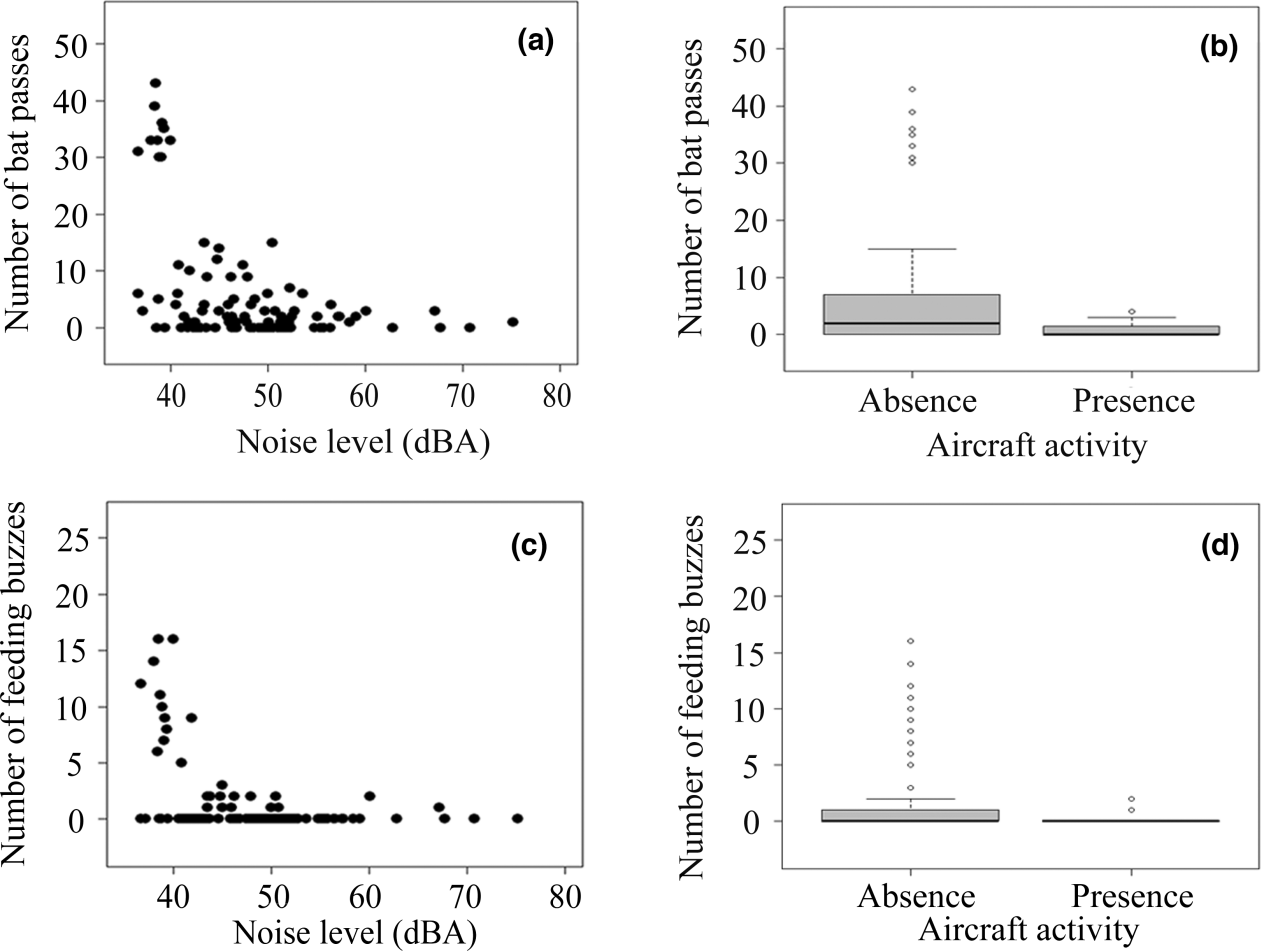
Relationship between airport noise and foraging activities of Japanese pipistrelle bats. (a) Airport noise level and number of bat passes. (b) Aircraft activity and number of bat passes. (c) Airport noise level and number of feeding buzzes. (d) Aircraft activity and number of feeding buzzes

Wind speed and moon phase were not retained in the best‐fitting GLMM for foraging activities of bats (Table S1). However, noise level (GLMM: β = −0.20, *z* = −2.77, *p* = .0056; Figure 2a), aircraft activity (GLMM: β = −7.88, *z* = −2.71, *p* = .0067; Figure 2b), habitat type (GLMM: β = 0.94, *z* = 3.74, *p* = .00018), and the interaction of noise level and aircraft activity (GLMM: β = 0.13, *z* = 2.43, *p* = .015; Table 1) were significant predictors of the number of bat passes. The nightly temperature (GLMM: β = 0.064, *z* = 1.80, *p* = .072) had no marked influence on the number of bat passes. Similarly, the number of feeding buzzes was predicted by noise level (GLMM: β = −0.49, *z* = −3.76, *p* = .00017; Figure 2c), aircraft activity (GLMM: β = −14.71, *z* = −2.96, *p* = .0031; Figure 2d), and the interaction of noise level and aircraft activity (GLMM: β = 0.28, *z* = 3.10, *p* = .0020; Table 1). The effects of habitat type (GLMM: β = 0.73, *z* = 1.51, *p* = .13) and nightly temperature (GLMM: β = 0.11, *z* = 1.90, *p* = .057) on the number of feeding buzzes were not significant.

**TABLE 1:** Effects of predictor variables on bat foraging activities based on the best‐fitting generalized linear mixed models

Foraging activities	Predictors	β	*Z*	*p*
Number of bat passes	Noise level	−0.20	−2.77	.0056
	Aircraft activity	−7.88	−2.71	.0067
	Habitat type	0.94	3.74	.00018
	Temperature	0.064	1.80	.072
	Noise level: Aircraft activity	0.13	2.43	.015
Number of feeding buzzes	Noise level	−0.49	−3.76	.00017
	Aircraft activity	−14.71	−2.96	.0031
	Habitat type	0.73	1.51	.13
	Temperature	0.11	1.90	.057
	Noise level: Aircraft activity	0.28	3.10	.0020

### 3.3 Echolocation call plasticity in foraging bats

**FIGURE 3:**
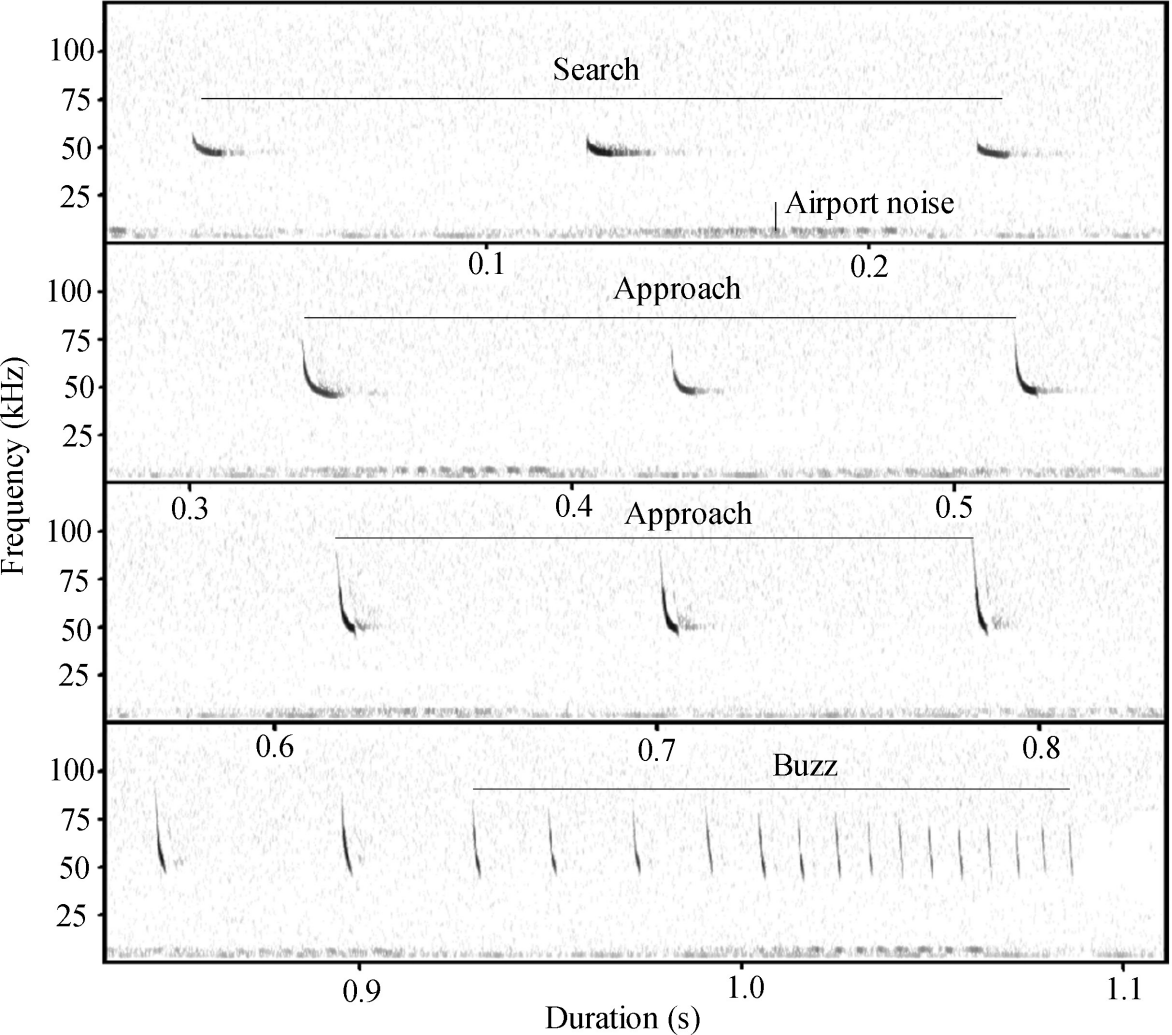
Spectrogram of airport noise and echolocation pulses emitted by Japanese pipistrelle bats. Call sequences were recorded while aircraft was preparing to land

**FIGURE 4:**
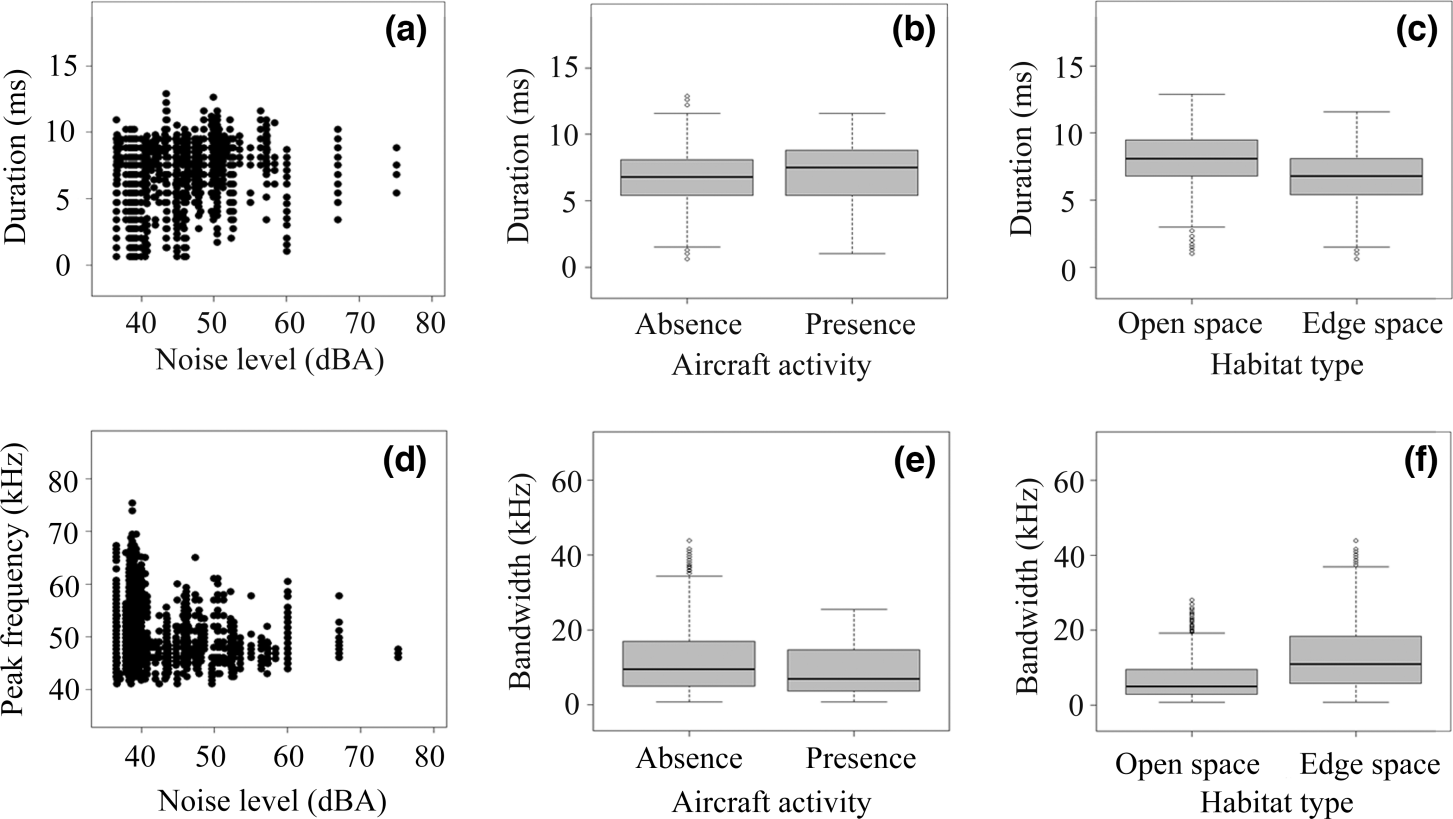
Echolocation pulse variation in Japanese pipistrelle bats. (a) Variation in pulse duration as a function of noise level. (b) Pulse duration under the presence and absence of aircraft. (c) Pulse duration in different habitat types. (d) Variation in peak frequency as a function of noise level. (e) Bandwidth under the presence and absence of aircraft. (f) Bandwidth in different habitat types

The average duration of echolocation pulses given by foraging bats was 6.71 ± 0.025 ms, average peak frequency was 49.49 ± 0.051 kHz, and average bandwidth was 11.55 ± 0.098 kHz (Table 2; Figure 3). The peak frequency of airport noise ranged from 1.40 to 10.90 kHz in the presence and absence of low‐flying aircraft, without spectral overlap with bat echolocation pulses (Table 2; Figure 3). Pulse duration was predicted by noise level (LMM: β = 0.49, *t* = 10.14, *p* < .0001; Figure 4a), aircraft activity (LMM: β = 18.63, *t* = 8.16, *p* < .0001; Figure 4b), habitat type (LMM: β = −0.83, *t* = −5.44, *p* < .0001; Figure 4c), nightly temperature (LMM: β = 19.16, *t* = 2.27, *p* = .024), wind speed (LMM: β = −272.97, *t* = −8.72, *p* < .0001), and the interaction of these factors (LMM: all *p* < .01; Table 3). Peak frequency was negatively associated with noise level (LMM: β = −9.08e‐7, *t* = −13.52, *p* < .0001; Figure 4d). Pulse bandwidth depended on aircraft activity (LMM: β = −0.29, *t* = −2.97, *p* = .0030; Figure 4e) and habitat type (LMM: β = 0.29, *t* = 6.23, *p* < .0001; Figure 4f).

**TABLE 2:** Spectro‐temporal parameters of bat echolocation vocalizations and airport noise

Parameters	Sound type			
	Pulse 1	Pulse 2	Noise 1	Noise 2
N_sample_	1048	5893	70	50
Pulse duration (ms)	8.01 ± 0.06	6.49 ± 0.03	–	–
Start frequency (kHz)	52.76 ± 0.20	59.35 ± 0.12	6.39 ± 0.26	10.63 ± 0.41
End frequency (kHz)	45.96 ± 0.06	46.97 ± 0.03	6.52 ± 0.29	10.04 ± 0.38
Peak frequency (kHz)	47.44 ± 0.09	49.85 ± 0.06	6.37 ± 0.30	8.93 ± 0.14
Bandwidth (kHz)	6.89 ± 0.17	12.37 ± 0.11	–	–

Note

Pulse 1: echolocation pulses in open space. Pulse 2: echolocation pulses in edge space. Noise 1: airport noise without the presence of aircraft. Noise 2: airport noise during the presence of aircraft. N_sample_: sample size.

**TABLE 3:** Effects of predictor variables on echolocation vocalizations in foraging bats based on the best‐fitting general linear mixed models

Parameters	Predictors	β	*t*	*p*
Pulse duration	Noise level	0.49	10.14	<.0001
	Aircraft activity	18.63	8.16	<.0001
	Habitat type	−0.83	−5.44	<.0001
	Temperature	19.16	2.27	.024
	Wind speed	−272.97	−8.72	<.0001
	Moon phase	11.51	0.17	.87
	Noise level × Aircraft activity	−0.33	−8.15	<.0001
	Temperature × Wind speed	19.72	10.88	<.0001
Peak frequency	Noise level	−9.08e‐7	−13.52	<.0001
Bandwidth	Aircraft activity	−0.29	−2.97	.0030
	Habitat type	0.29	6.23	<.0001

## 4 DISCUSSION

Despite an increasing interest in noise pollution (Barber et al., 2010; Finch et al., 2020; Luo, Goerlitz, et al., 2015; Luo, Siemers, et al., 2015; Senzaki et al., 2020), experimental evidence in support of deterrent effects of airport noise on foraging behavior in bats is still lacking. In this study, we used *P*. *abramus* foraging near a municipal airport as a model to address this question. Our acoustic monitoring around the runway indicated that airport noise intensity and aircraft activity were negative determinants of bat passes and feeding buzzes, two crucial indicators of feeding activities in *P*. *abramus*. The negative relationship between airport noise and bat feeding activities was robust, even after incorporating information concerning habitat structure and other environmental factors. Although airport noise showed no apparent spectral overlap with echolocation vocalizations in foraging bats, the intensity of airport noise and aircraft activity was tightly linked to pulse duration and spectral parameters. Collectively, these results provide correlative evidence supporting the hypothesis that airport noise disturbs foraging behavior of pipistrelle bats.

As expected, the number of bat passes and that of feeding buzzes declined with noise level around the runway, especially under the presence of aircraft. This suggests that high‐level airport noise can impede foraging behavior of *P*. *abramus*. A similar phenomenon has been documented in some other wild animals. Within the order Chiroptera, Brazilian free‐tailed bats reduce evening foraging activities in noisy areas with compressor stations (Bunkley et al., 2015). The feeding success of Daubenton’s bats and pallid bats (*Antrozous pallidus*) declined when experimentally exposed to road traffic noises and compressor noises, respectively (Bunkley & Barber, 2015; Luo, Siemers, et al., 2015). The greater mouse‐eared bats responded to traffic noise from the highway by increasing search time for food in a dose–response fashion as the noise level gradually increased (Siemers & Schaub, 2011). In great tits, the ratio between vigilance duration and foraging bouts was affected by the noise level of commercial aircraft (Klett‐Mingo et al., 2016). In prairie dogs (*Cynomys ludovicianus*), traffic noise inhibited their aboveground foraging activities and enhanced their vigilance responses (Shannon et al., 2014). The harmful effects of ship noise on foraging‐related activities have also been observed in aquatic animals such as humpback whales (*Megaptera novaeangliae*) (Blair et al., 2016), three‐spined sticklebacks (*Gasterosteus aculeatus*) (Voellmy et al., 2014), and harbor porpoises (*Phocoena phocoena*) (Wisniewska et al., 2018). These findings confirm that anthropogenic noise serves as a negative factor underlying foraging behavior in most vertebrate groups.

How does airport noise affect foraging behavior in *P*. *abramus*? According to previous research, anthropic noise may disturb foraging activities of vocalizing animals via three main mechanisms, i.e., masking (Brumm & Slabbekoorn, 2005; Siemers & Schaub, 2011; Slabbekoorn & Peet, 2003), distraction (Chan et al., 2010), and aversion or stress (Luo, Siemers, et al., 2015; Wright et al., 2007). The acoustic masking hypothesis proposes that anthropogenic noise can hinder the detection and discrimination of target sounds if they overlap spectrally and temporally (Brumm & Slabbekoorn, 2005). Supporting this hypothesis, foraging success of gleaning greater mouse‐eared bats is comparatively low when traffic noises mask rustling sounds generated by prey movement, particularly at high noise levels (Siemers & Schaub, 2011). The acoustic masking hypothesis predicts that airport noise would have negligible effects on bat passes and feeding buzzes in *P*. *abramus*, given that frequency parameters of airport noise were significantly lower than those of ultrasonic pulses and associated prey echoes. In contrast, the acoustic distraction hypothesis emphasizes that anthropogenic noise acts as a distracting signal, diverting the limited attention and processing power of animals from feeding tasks to noise stimuli (Chan et al., 2010; Luo, Siemers, et al., 2015). Indeed, gleaning pallid bats (*Antrozous pallidus*) consistently increase search time and repetition rates of echolocation vocalizations under exposure to white noises that do or do not overlap spectrally with prey‐generated sound cues (Allen et al., 2021). As per acoustic distraction hypothesis, the airport noise level should scale positively with the number of bat passes that correspond to feeding efforts but negatively with the number of feeding buzzes that represent successful prey captures. Finally, the acoustic aversion hypothesis underscores that anthropogenic noise acts as an aversive stimulus for foraging animals, eliciting avoidance responses regardless of the extent of spectral overlap between target sounds and noise stimuli. Consistent with the prediction of the acoustic aversion hypothesis, our acoustic survey revealed that the level of spectrally non‐overlapping airport noise was a negative predictor for bat passes and feeding buzzes, a sign of noise avoidance as observed in Daubenton’s bats, common pipistrelles, and soprano pipistrelles (Finch et al., 2020; Luo, Siemers, et al., 2015). Moreover, physiological experiments corroborated the suggestion that the audiogram of *P*. *abramus* exhibited a broad U‐shape covering the frequency range from 4 to 80 kHz, indicating that low‐frequency airport noise reaches the hearing range of this species. Therefore, noise aversion appears to account for the negative effects of airport noise on foraging behavior of *P*. *abramus*.

The spectro‐temporal characteristics of echolocation pulses in *P*. *abramus* were dependent on noise level and associated aircraft activity (Figure S2) around the runway. Three potential explanations may be offered for this phenomenon. First, modification in echolocation vocalizations may be an adaptive strategy against noise interference at first sight. Many vocalizing animals, including echolocating bats, mitigate masking effects from anthropogenic noise by increasing sound frequency, amplitude, repetition rate, or a combination of these parameters (Kunc & Schmidt, 2021; Roca et al., 2016). In greater horseshoe bats (*Rhinolophus ferrumequinum*), for example, both pulse amplitude and frequency parameters are raised when broadband noises mask the dominant frequency component of echolocation pulses (Hage et al., 2013). Following experimental manipulation of synthesized noises that covered the spectrum of echolocation signals, pale spear‐nosed bats (*Phyllostomus discolor*) increased pulse amplitude, duration, and redundancy (an indicator of repetition rate) with increasing noise level, largely improving signal detectability under noisy conditions (Luo, Goerlitz, et al., 2015). However, we found that the level of spectrally non‐overlapping airport noise was also a significant predictor of pulse duration and peak frequency in *P*. *abramus*, indicating that pulse variation is not an adaptive response to counteract noise interference. Second, some bats show individual differences in echolocation pulse adjustments and feeding performance in the presence of anthropogenic noise, including free‐tailed bats (*Tadarida brasiliensis*) (Tressler & Smotherman, 2009), Daubenton’s bats (Luo, Siemers, et al., 2015), and pale spear‐nosed bats (Gomes & Goerlitz, 2020). In this case, it is expected that a small number of *P*. *abramus* within the population can tolerate high levels of airport noise and perform foraging activities at noisy sites. Therefore, individual variation in noise sensitivity together with individual‐specific echolocation pulses may explain the observed relationship between airport noise level and echolocation vocalizations in *P*. *abramus*. Third, acute and chronic noises can serve as an aversive stimulus eliciting physiological stress in some birds and mammals, thereby causing increases in hormone concentrations, cardiovascular activities, and respiration rates (Kight & Swaddle, 2011; Kleist et al., 2018; Wright et al., 2007). Given the close link between respiration and pulse emission (Lancaster & Speakman, 2001; Speakman & Racey, 1991), it is likely that the observed relationship between airport noise and echolocation vocalizations in *P*. *abramus* is indicative of a byproduct of physiological stress. Further research is needed to explore the mechanisms behind the impacts of airport noise on echolocation signal design in bats.

*P*. *abramus* showed considerable plasticity in echolocation vocalizations in different foraging habitats, a result that is in line with previous findings (Kalko & Schnitzler, 1993; Siemers et al., 2001; Wang et al., 2014). In open space, echolocation pulses given by foraging *P*. *abramus* were characterized by relatively long duration, low frequency, and narrow bandwidth. However, *P*. *abramus* shortened pulse duration but raised bandwidth and frequency parameters while foraging in edge space. This is not surprising, given that echolocating bats can adjust echolocation vocalizations as a function of habitat clutter level (Kalko & Schnitzler, 1993; Schnitzler et al., 2003). When foraging in uncluttered space, an increase in pulse duration can improve signal redundancy and thus detectability in echolocating bats, albeit very long pulses cause temporal overlap between prey echo and the emitted signal (Luo, Goerlitz, et al., 2015). The low‐frequency pulses are beneficial for long‐range detection of insect prey due to less attenuation in air (Lawrence & Simmons, 1982; Luo, Goerlitz, et al., 2015). The narrowband pulses facilitate concentrating spectral energy, and their echoes can rapidly activate neuronal filters that are responsible for prey detection (Kalko & Schnitzler, 1998). By contrast, echolocating bats face a high risk of auditory masking while foraging in edge space, since prey echo can be interfered by the emitted pulses and clutter echoes (Kalko & Schnitzler, 1998; Schnitzler et al., 2003). To mitigate auditory masking, low duty cycle (LDC) bats reduce pulse duration to avoid the overlap between prey echo and interfering signals, whereas high duty cycle (HDC) bats employ Doppler shift compensation to maintain echo frequency within the sensitive frequency range of the auditory fovea (Fenton, 2013; Schnitzler et al., 2003). In addition, broadband pulses cover a multitude of wavelengths that can ensonify many reflecting surfaces, and thus confer advantages in prey classification and background discrimination in edge habitats (Siemers & Schnitzler, 2004). Broadband pulses can also activate more neuronal filters compared with narrowband signals, thereby enhancing the accuracy of range and angle determination for echolocating bats (Kalko & Schnitzler, 1998). Overall, these findings indicate that the plasticity of echolocation vocalizations in bats represents an adaptive response to foraging ecology.

To summarize, our acoustic survey demonstrated that airport noise can interfere with foraging behavior of Japanese pipistrelle bats, even if it does not overlap spectrally with echolocation pulses and associated prey echoes. Following an increase in the noise level at sampling sites around the runway, the number of bat passes and that of feeding buzzes declined. This implies that noise aversion, instead of noise masking and distraction, is involved in the negative effects of airport noise on foraging behavior of *P*. *abramus*. *P*. *abramus* also adjust temporal and spectral parameters of echolocation pulses according to foraging habitats, indicating that echolocation is a flexible active sensory mode enabling echolocating bats to search and orient toward prey in complex environments. The observed relationship between spectrally non‐overlapping airport noise and echolocation pulse parameters in *P*. *abramus* may be attributed to individual variation in call design and noise sensitivity, indirect effect of physiological stress, or both. These results highlight that adverse impacts of anthropogenic noise on foraging activities are also evident in pipistrelle bats, a common synanthropic species living in artificial ecosystems. Combined with previous research (Buxton et al., 2017; Francis & Barber, 2013; Luo, Goerlitz, et al., 2015; Luo, Siemers, et al., 2015), our findings offer implications for strengthening noise assessment and managements in foraging habitats utilized by bats.

## AUTHOR CONTRIBUTION

**Weiwei Wang:** Conceptualization (supporting); Investigation (equal); Methodology (equal); Writing – review & editing (equal). **Huimin Gao:** Investigation (supporting); Resources (supporting); Writing – review & editing (supporting). **Chengrong Li:** Investigation (supporting); Methodology (supporting); Resources (supporting). **Yingchun Deng:** Investigation (supporting); Methodology (supporting); Resources (supporting). **Daying Zhou:** Investigation (supporting); Methodology (supporting); Resources (supporting). **Yaqi Li:** Investigation (supporting); Methodology (supporting); Resources (supporting). **Wenyu Zhou:** Methodology (supporting); Resources (supporting). **Bo Luo:** Conceptualization (equal); Methodology (equal); Resources (equal); Writing – review & editing (equal). **Haiying Liang:** Investigation (supporting); Methodology (supporting). **Wenqin Liu:** Methodology (supporting); Resources (supporting). **Pan Wu:** Investigation (supporting); Methodology (supporting). **Wang Jing:** Investigation (supporting); Methodology (supporting). **Jiang Feng:** Conceptualization (equal); Resources (equal).

## CONFLICT OF INTEREST

The authors declare no competing interests.

## Supporting information

Supplementary Material

## Data Availability

The dataset used in this study is publicly available in Dyrad Digital Repository (https://doi.org/10.5061/dryad.3ffbg79mb).
