# Supplementary material for: Airport noise disturbs foraging behavior of Japanese pipistrelle bats
Source: Ecol Evol. 2022 Jun 12;12(6):e8976. doi: 10.1002/ece3.8976 (PMC9189338; doi:10.1002/ece3.8976)
Supplement: Supplementary file 1 — Supplementary Material [file ECE3-12-e8976-s001.docx]

**
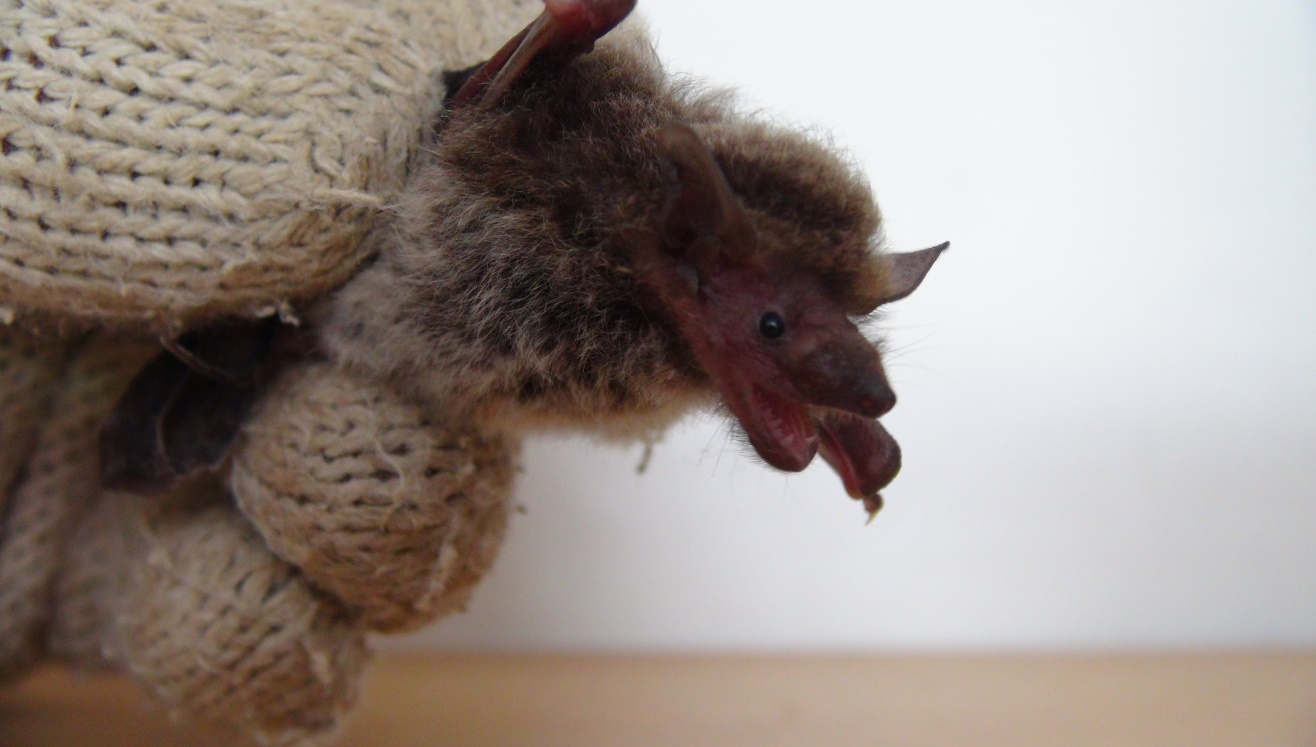
**

**FIGURE S1** The photograph of a male Japanese pipistrelle bat captured around the airport

**TABLE S1** The first five alternative generalized and general linear mixed models (GLMM and LMM) in this study

| Dependent variable | Model | AICc | ∆AICc | Predictors |
| --- | --- | --- | --- | --- |
| Number of bat passes | **GLMM 1** | **517.50** | **_** | **Noise level + Aircraft activity + Habitat type + Temperature + Noise level ×** **Aircraft activity** |
|  | GLMM 2 | 518.39 | 0.89 | Noise level + Aircraft activity + Habitat type + Noise level × Aircraft activity |
|  | GLMM 3 | 518.64 | 1.14 | Noise level + Aircraft activity + Habitat type + Temperature + Moon phase + Noise level × Aircraft activity |
|  | GLMM 4 | 518.69 | 1.19 | Aircraft activity + Habitat type |
|  | GLMM 5 | 518.89 | 1.39 | Noise level + Aircraft activity + Habitat type + Temperature + Wind speed + Noise level × Aircraft activity |
| Number of feeding buzzes | **GLMM 1** | **240.75** | **_** | **Noise level + Aircraft activity + Habitat type + Temperature + Noise level × Aircraft activity** |
|  | GLMM 2 | 240.79 | 0.03 | Noise level + Aircraft activity + Temperature + Noise level × Aircraft activity |
|  | GLMM 3 | 241.36 | 0.61 | Noise level + Aircraft activity + Temperature + Wind speed + Noise level × Aircraft activity |
|  | GLMM 4 | 241.45 | 0.70 | Noise level + Aircraft activity + Habitat type + Temperature + Moon phase + Noise level × Aircraft activity |
|  | GLMM 5 | 241.69 | 0.94 | Noise level + Aircraft activity + Habitat type + Temperature + Wind speed + Noise level × Aircraft activity |
| Pulse duration | **LMM1** | **35578.67** | **_** | **Noise level + Aircraft activity + Habitat type + Temperature + Wind speed + Moon phase + Noise level × Aircraft activity + Temperature × Wind speed** |
|  | LMM 2 | 35604.31 | 25.64 | Noise level + Aircraft activity + Temperature + Wind speed + Moon phase + Noise level × Aircraft activity + Temperature × Wind speed |
|  | LMM 3 | 35635.14 | 56.47 | Noise level + Habitat type + Temperature + Wind speed + Moon phase + Temperature × Wind speed |
|  | LMM 4 | 35636.72 | 58.05 | Noise level + Aircraft activity + Habitat type + Temperature + Wind speed + Moon phase + Temperature × Wind speed |
|  | LMM 5 | 35668.95 | 90.28 | Noise level + Temperature + Wind speed + Moon phase + Temperature × Wind speed |
| Peak frequency | **LMM 1** | **-126514.52** | **_** | **Noise level** |
|  | LMM 2 | -126501.65 | 12.88 | Noise level + Aircraft activity |
|  | LMM 3 | -126500.83 | 13.69 | Noise level + Habitat type |
|  | LMM 4 | -126491.66 | 22.86 | Noise level + Moon phase |
|  | LMM 5 | -126491.39 | 23.13 | Noise level + Wind speed |
| Bandwidth | **LMM 1** | 22261.25 | _ | **Aircraft activity + Habitat type** |
|  | LMM 2 | 22264.65 | 3.40 | Aircraft activity + Habitat type + Moon phase |
|  | LMM 3 | 22264.81 | 3.57 | Noise level + Habitat type |
|  | LMM 4 | 22265.22 | 3.97 | Habitat type |
|  | LMM 5 | 22266.58 | 5.33 | Aircraft activity + Habitat type + Wind speed |

AICc: Akaike’s information criterion corrected for small sample size. ΔAICc: the difference between the alternative model and best-fitting model.

**TABLE S2** Foraging activities of Japanese pipistrelle bats and noise level per site

| Sites | Number of bat passes | Number of feeding buzzes | Noise level (dBA) |
| --- | --- | --- | --- |
| Site 1 | 7.80 ± 1.06 | 1.20 ± 0.55 | 41.74 ± 1.13 |
| Site 2 | 4.90 ± 1.78 | 0.70 ± 0.26 | 49.87 ± 1.84 |
| Site 3 | 5.70 ± 1.16 | 0.50 ± 0.27 | 49.81 ± 1.43 |
| Site 4 | 1.00 ± 0.47 | 0.10 ± 0.10 | 52.44 ± 2.52 |
| Site 5 | 1.00 ± 0.45 | 0.30 ± 0.21 | 51.23 ± 2.54 |
| Site 6 | 1.60 ± 0.65 | 0.00 ± 0.00 | 55.38 ± 3.23 |
| Site 7 | 0.30 ± 0.21 | 0.00 ± 0.00 | 47.84 ± 1.86 |
| Site 8 | 1.00 ± 0.54 | 0.00 ± 0.00 | 48.96 ± 1.43 |
| Site 9 | 0.90 ± 0.28 | 0.90 ± 0.90 | 47.69 ± 1.25 |
| Site 10 | 0.00 ± 0.00 | 0.00 ± 0.00 | 48.86 ± 1.97 |
| Site 11 | 34.30 ± 1.30 | 10.90 ± 1.13 | 38.63 ± 0.28 |


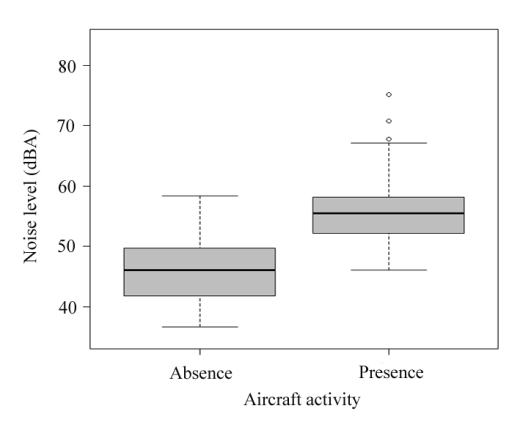


**FIGURE S2** The effect of aircraft activity on noise level among sampling sites. The statistical significance is evident based on one-way ANOVA analysis (*F*_1, 108_ = 77.20, *p* < 0.0001)
